# The ANTOP study: focal psychodynamic psychotherapy, cognitive-behavioural therapy, and treatment-as-usual in outpatients with anorexia nervosa - a randomized controlled trial

**DOI:** 10.1186/1745-6215-10-23

**Published:** 2009-04-23

**Authors:** Beate Wild, Hans-Christoph Friederich, Gaby Gross, Martin Teufel, Wolfgang Herzog, Katrin E Giel, Martina de Zwaan, Henning Schauenburg, Carmen Schade-Brittinger, Helmut Schäfer, Stephan Zipfel

**Affiliations:** 1 Department of Psychosomatic and General Internal Medicine, Medical University Hospital, Heidelberg, Germany; 2 Department of Psychosomatic Medicine and Psychotherapy, Medical University Hospital, Tuebingen, Germany; 3 Department of Psychosomatic Medicine and Psychotherapy, Medical University Hospital, Erlangen, Germany; 4 Coordinating Centre for Clinical Trials (KKS), Philipps-University, Marburg, Germany; 5 Institute of Medical Biometry and Epidemiology, Philipps-University, Marburg, Germany

## Abstract

**Background:**

Anorexia nervosa is a serious eating disorder leading to high morbidity and mortality as a result of both malnutrition and suicide. The seriousness of the disorder requires extensive knowledge of effective treatment options. However, evidence for treatment efficacy in this area is remarkably weak. A recent Cochrane review states that there is an urgent need for large, well-designed treatment studies for patients with anorexia nervosa. The aim of this particular multi-centre study is to evaluate the efficacy of two standardized outpatient treatments for patients with anorexia nervosa: focal psychodynamic (FPT) and cognitive behavioural therapy (CBT). Each therapeutic approach is compared to a "treatment-as-usual" control group.

**Methods/Design:**

237 patients meeting eligibility criteria are randomly and evenly assigned to the three groups – two intervention groups (CBT and FPT) and one control group. The treatment period for each intervention group is 10 months, consisting of 40 sessions respectively. Body weight, eating disorder related symptoms, and variables of therapeutic alliance are measured during the course of treatment. Psychotherapy sessions are audiotaped for adherence monitoring. The treatment in the control group, both the dosage and type of therapy, is not regulated in the study protocol, but rather reflects the current practice of established outpatient care. The primary outcome measure is the body mass index (BMI) at the end of the treatment (10 months after randomization).

**Discussion:**

The study design surmounts the disadvantages of previous studies in that it provides a randomized controlled design, a large sample size, adequate inclusion criteria, an adequate treatment protocol, and a clear separation of the treatment conditions in order to avoid contamination. Nevertheless, the study has to deal with difficulties specific to the psychopathology of anorexia nervosa. The treatment protocol allows for dealing with the typically occurring medical complications without dropping patients from the protocol. However, because patients are difficult to recruit and often ambivalent about treatment, a drop-out rate of 30% is assumed for sample size calculation. Due to the ethical problem of denying active treatment to patients with anorexia nervosa, the control group is defined as "treatment-as-usual".

**Trial registration:**

Current Controlled Trials ISRCTN72809357

## Background

Anorexia nervosa (AN) is a serious eating disorder marked by pronounced, self-induced underweight. Diagnostic criteria regarding weight are defined as maintenance of body weight < 85% of expected weight, or a body mass index (BMI) of < 17.5 kg/m^2 ^(DSM-IV; ICD-10). Weight reduction is induced by abnormal food restriction with or without purging behaviours such as self-induced vomiting, compulsive exercise, and/or use of laxatives and diuretics; other features include an intense fear of gaining weight and a distorted body image. The average prevalence rate of AN among young females is estimated to be 0.3% whereas the prevalence in men is about ten times lower [1]. Several long-term studies document the seriousness of anorexia nervosa [2], concluding that about half the patients fully recover from anorexia nervosa whereas a third improve and 20% remain chronically ill [3]. In a 21-year follow-up study of 84 patients, the crude mortality rate was 16% with a standardized mortality rate of 9.8 [4,5].

In regard to treatment efficacy, the evidence to date is judged to be weak [6]. In the guidelines of the British National Institute of Clinical Excellence [7], treatment recommendations for anorexia nervosa are based solely on expert reports and clinical experiences because clinical studies of good quality are absent. To date, only a few minor randomized controlled trials of individualized adult outpatient therapy for anorexia nervosa exist [8,9]. The trials are barely comparable as different types of psychotherapy and control groups were used. For instance, results of the study of Dare et al. [10] found no difference among the three specific therapies but showed that focal psychotherapy and family therapy were more effective in inducing weight gain than the control treatment (low-contact outpatient management). One trial found that a non-specific therapy was favoured over the two specific psychotherapies (cognitive behaviour therapy and interpersonal psychotherapy) [9]. However, no specific treatment was identified that was consistently superior to any other specific approach. Additionally, sample size in all studies was very small. Hay et al. [8], in their systematic review, therefore concluded that there is an urgent need for large multi-centre trials of commonly used psychotherapies for adults with anorexia nervosa.

In spite of an absence of evidence, many treatment approaches exist in clinical practice. In Germany, two commonly used treatment approaches are psychodynamic psychotherapy and cognitive behavioural therapy. In patients with anorexia nervosa, psychodynamic therapy addresses un- or pre-conscious conflicts that underlie the disorder. A basic approach of psychodynamic treatment in AN is to attend to the dysfunctional shaping of relationships and structural deficits in regard to the regulation of emotions that are relevant to the disordered eating behaviour [11]. Focal psychodynamic psychotherapy (FPT) was developed as a standardised form of time-limited psychodynamic psychotherapy [12].

In the treatment of eating disorders, the aims of cognitive behavioural therapy (CBT) focus on the modification of behaviours and cognitions that maintain the existing behaviour. Main foci are the modification of automatic negative thoughts and dysfunctional assumptions that relate to food, eating, weight, and body shape [13,14].

The aim of the ANTOP (Anorexia Nervosa Treatment of OutPatients) study is the examination of the efficacy of two standardised outpatient treatment programs for patients with anorexia nervosa – focal psychodynamic (FPT) and cognitive behavioural therapy (CBT) – in comparison to treatment-as-usual (TAU).

## Methods

### Study centres

This particular study is being carried out at 10 trial sites. Participating centres are the Departments of Psychosomatic Medicine and Psychotherapy of the Universities of Dortmund/Bochum, Erlangen, Essen, Freiburg, Hamburg, Heidelberg, München, Münster, Tübingen and Ulm. Patients are recruited in the outpatient departments of the hospitals. To increase the number of eligible patients, the study is advertised in local as well as regional media.

### Participants

Inclusion and exclusion criteria are listed in Table 1. According to these criteria, the sample of patients included in the study is representative of patients who qualify for treatment in an outpatient setting who suffer from either anorexia nervosa or subsyndromal anorexia nervosa.

**Table 1:** Inclusion and exclusion criteria of the ANTOP study

Inclusion criteria	• AN and subsyndromal AN (lacking one diagnostic criterion according to DSM IV such as amenorrhea or weight phobia)
	• Female
	• Age 18 years or older
	• 15.0 < BMI < 18.5 kg/m^2^
	• written informed consent of the patient
Exclusion criteria	• Current substance abuse
	• Current medication with neuroleptics
	• Current suicidal ideation
	• Psychotic disorder
	• Bipolar disorder
	• Serious unstable medical problems/complications
	• Ongoing psychotherapy
	• Pregnancy
	• Clinically relevant cardiac arrhythmia
	• Primary somatic disease

### Interventions

Treatments for both the FPT and CBT groups are individual outpatient interventions, and are based on standardised treatment manuals. In these groups, the therapy is comprised of 40 sessions over a period of 10 months. This fixed time frame was chosen in accordance with the usual practice of the German health care system. CBT and FPT are time-limited treatment approaches that have a main advantage "to concentrate the mind of both the patient and the therapist" [13]. At the same time, patients with AN need a high intensity of treatment. Fairburn suggests a total of 40 CBT sessions for AN patients with a BMI between 15.0 and 17.5; this corresponds well to our study sample.

**Figure 1:**
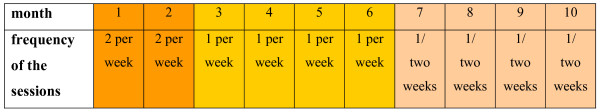
**Frequency of therapy sessions in the intervention conditions**. It has to be noted that a treatment period of 40 sessions does not equal always 10 months. We therefore defined the measurement point T2 as 10 months after inclusion in the study (± 2 weeks).

The frequency of the sessions is shown in Figure 1:

FPT and CBT sessions are audiotaped to monitor adherence to treatment manuals. Every ninth audiotape is checked by monitors in Tübingen (CBT) or Heidelberg (FPT) to ensure treatment integrity. To avoid contamination, the two treatments are provided by different therapists. Treatment providers – psychologists or medical doctors – are trained for a minimum of three years in the respective psychotherapy methods and receive additional training in the use of the manual. At every fourth session, experienced supervisors provide supervision to the therapists.

The psychodynamic treatment plan can roughly be subdivided into three treatment phases. The first phase focuses primarily on therapeutic alliance, ego-syntonicity of the disorder, and self-esteem. In the second phase of treatment, the main focus is placed on the association between interpersonal relationships and eating (anorectic) behaviour. In the last phase, relevant aspects are the transfer to everyday life, anticipation of treatment termination, and parting.

The cognitive behavioural treatment plan also consists of several treatment modules. The manual is based on the work of Fairburn [13]. Work sheets are adapted to Legenbauer & Vocks [15]. At each session, homework, work sheets, and exercises are assigned. The treatment plan focuses on patients' education about underweight and starvation as well as on the initiation and maintenance of regular dietary habits and weight gain. Enhancement of self-efficacy and self-monitoring are crucial elements of the entire treatment process. Further details of the two interventions are described in the treatment manuals [16].

The patients that are randomly assigned to the TAU control group receive support for further therapy planning. The patients receive a list of established outpatient therapists who practice psychotherapy in accordance with the general German psychotherapy guidelines. This is the standard approach for the allocation of patients to treatment in the German health care system with its sectoral separation of institutions that provide inpatient care and of individual practitioners that provide outpatient care. In the study protocol, the treatment (dosage and kind of therapy) in the TAU arm is not regulated in order to get a naturalistic and representative assessment of both the usual outpatient care and the psychotherapeutic treatment for patients with AN.

### Measurement time points

**Figure 2:**
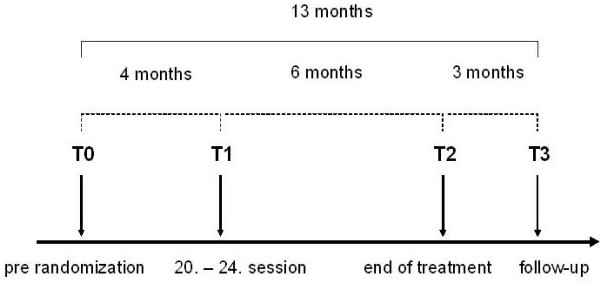
**Time points of measurement in the ANTOP study**.

The time points of measurement are shown in Figure 2.

A three-month follow-up is included in the first funding period of this study. Funding for a one-year follow-up has been applied for.

### Objectives and hypotheses

The objective of the current study is to assess whether two psychotherapeutic interventions are more effective than treatment-as-usual for the treatment of AN in adults. The primary hypothesis has two parts: Part one states that compared to treatment-as-usual, the specific psychotherapeutic outpatient intervention FPT shows a better outcome regarding the BMI at the end of treatment (T2). Part two states that compared to treatment-as-usual, the specific psychotherapeutic outpatient intervention CBT shows a better outcome regarding the BMI at the end of treatment (T2). That is, the null hypotheses tested in the confirmatory analysis are  and  where *μ *denotes the expected BMI value at the end of the respective treatment (T2), adjusted for the pre-randomization baseline BMI (T0).

Secondary (explorative) hypotheses state: (1) that the specific interventions (FPT, CBT) do not differ in terms of BMI increase at the end of treatment (T2); (2) that compared to TAU, FPT shows a better outcome at the end of treatment (T2) in regard to both a gain in quality of life and other psychosocial variables; (3) that compared to TAU, CBT shows a better outcome at the end of treatment in regard to both a gain in quality of life and other psychosocial variables.

### Outcomes

The primary outcome measure is the body mass index (BMI) at the end of the treatment (10 months after randomization). Secondary outcome measures are: the Morgan-Russell-criteria [17,18]; general axis I psychopathology (SCID I) [19]; eating disorder specific psychopathology (SIAB-Ex; Eating Disorder Inventory-2) [20,21]; severity of depressive comorbidity (PHQ-9) [22]; and quality of life according to the SF-36 [23]. The therapeutic alliance is assessed by the Helping Alliance Questionnaire (HAQ) [24]. Table 2 shows the assessments and outcome measurements at the different time points in the study.

**Table 2:** Assessment of secondary outcome measures within the ANTOP study

	**Assessment**	
**Point in study**	**Expert rating**	**Self-report**

T0	SCID-I SIAB-Ex Morgan-Russell Outcome Assessment Schedule	EDI-2 PHQ-D SF-36
	
T1	Morgan-Russell Outcome Assessment Schedule	
	
T2	SCID-I SIAB-Ex Morgan-Russell Outcome Assessment Schedule	
	
T3	Morgan-Russell Outcome Assessment Schedule	

Monthly (during the treatment phase)		HAQ

EDI-2: Eating Disorder Inventory-2; HAQ: Helping Alliance Questionnaire; PHQ-D: German version of the Patient Health Questionnaire; SCID-I: Structured Clinical Interview for DSM-IV Axis I Disorders; SF-36: Questionnaire on health-related quality of life; SIAB-Ex: Structured expert interview for anorectic and bulimic eating disorders

Outcome assessments are either self-reported or performed by an independent blinded observer. Assessors are trained at the beginning of the study. Blinding of the participants is not possible.

### Sample size calculation

Estimates of a clinically relevant effect size were derived from two randomized controlled studies that evaluated the effects of FPT and CBT in outpatients with AN [10,25]. Based on these findings, it was assumed that the different interventions would result in an improvement of 1.0 BMI-points compared to the TAU-group, with a pooled standard deviation of 1.7 BMI-points (effect size = 0.59). As the hypothesis for the primary efficacy endpoint comprises two statistical tests, namely FPT vs. TAU and CBT vs. TAU, the nominal α-level was set to 2.5% (two-sided) in order to restrict the Type 1 error level to 5%. Calculations were done to detect an effect size of 0.59 for a comparison power of 80%. Using a two-sample t-test, a sample size of 55 patients per treatment arm was required, resulting in a total sample size of n = 3*55 = 165. Considering a non-compliance and loss to follow up rate of 30% in total, a minimum of 237 patients must be included in the study.

### Randomization

Subjects are randomly assigned to the three groups. Randomization is stratified by duration of AN, resulting in two stratification groups. To ensure that allocation to the various groups is concealed, a centralized randomization is carried out by the Coordination Centre for Clinical Trials (KKS) in Marburg, Germany. Eligibility assessment, obtaining informed consent, and enrolling the patient in the trial is done by the respective study centres. The information data of the enrolled patient is transferred to the KKS by fax.

### Statistical analysis

At the conclusion of the study, intention to treat analyses will be done. Missing values will be replaced by using the Last Observation Carried Forward (LOCF) method. Additional sensitivity analyses using further imputation methods will also be done. For the primary analysis, both experimental treatment groups (FPT and CBT) will be compared with the TAU control group. The primary outcome – BMI at T2 – will be analysed using a mixed modelling approach. The grand mean, the treatment effects, and the stratification variable will be designed as fixed effects. The centre effect will be modelled as a random effect. The baseline BMI is entered as covariate in the model. The model can be written as:



where *Y*_*ijkl *_denotes the BMI at T2 on the *l*th patient  on treatment *i *(*i *= 1,2,3) in stratum *j *(*j *= 1,2) in centre *k *(*k *= 1,...10), μ denotes the overall mean, *τ*_*i *_denotes the effect of the *i*th treatment, *α*_*j *_denotes the effect of the *j*th stratum, *ζ*_*k *_denotes the random effect of the *k*th centre (*ζ*_*j *_~N(0, )), β denotes the baseline covariate effect, *X*_*l *_denotes the baseline BMI on the *l*th patient, and *ε*_*ijkl *_denotes a residual error with *ε*_*ijkl *_~ N(0, ). The parameters of interest in this confirmatory analysis are the mean of the control group *μ *+ *τ*_1 _and the contrasts of the means *τ*_2 _- *τ*_1 _and *τ*_3 _- *τ*_1 _(that indicate the treatment effects of FPT and CBT).

The secondary outcome measures will also be analysed using mixed models. No interim or subgroup analyses are planned.

### Safety aspects

In AN patients, food restriction can lead to life threatening starvation that requires inpatient medical monitoring and treatment. As food restriction and severe weight loss are central to the psychopathology of AN, an intermittent inpatient treatment up to four weeks is not considered a Serious Adverse Event (SAE). All other fatal or life-threatening events are defined as SAE. SAEs have to be reported immediately to the principal investigator and the central ethics committee. All adverse events are documented and reported to the principal investigator. An Independent Safety Monitoring Board was implemented at the outset.

### Medical complications

If, over the course of a two-week measurement period, a patient of an intervention group (CBT, FPT) has a BMI < 14, the outpatient treatment is interrupted and the patient is hospitalized. If the inpatient treatment leads to an improvement within four weeks (BMI ≥ 14), the outpatient treatment is resumed; if there is no improvement, the patient is withdrawn from the intervention treatment. Patients of the TAU-condition are advised to see their general practitioner (GP) on a regular monthly basis to monitor weight and possible medical complications. The study staff is in contact with the GPs to arrange more intensive treatment for the patient if necessary. Moreover, a control of relevant blood parameters is also part of the diagnostic measurement points (T0 – T3).

In the event of medical complications such as malnutrition or the manifestation of a severe psychiatric disorder, short-term hospitalization should be considered. For further clinical decisions, the guidelines of the American Psychiatric Association 2006  as well as the recommendations of J. Treasure  should be considered.

## Discussion

The ANTOP study aims to provide new evidence about the efficacy of two different therapy approaches in the outpatient treatment of anorexia nervosa: focal psychodynamic therapy and cognitive-behavioural therapy. The design of the study overcomes the disadvantages of previous studies in that it provides a randomized controlled design, a large sample size, adequate inclusion criteria, an adequate treatment protocol, and a clear separation of intervention conditions.

This is the first multi-centre trial to include a sample size of adults with AN that is large enough to detect a reasonable effect size. The assumed drop-out rate of 30% is similar to the drop-out rates of previous smaller trials. As the assumed drop-out rate is quite high, special attention must be given to the method of missing data imputation in the data analysis. The ANTOP study is further distinguished from previous studies in that an adequate definition of inclusion criteria has been formulated. For instance, McIntosh et al. [9] included patients with an average BMI of 17.3; this made it difficult to generalize the results to patients with AN. In the ANTOP study, the inclusion criteria of having AN (or subsyndromal AN) and a BMI between 15.0 and 18.5 kg/m^2 ^ensure that the sample of patients included in the study is representative. Furthermore, in contrast to the study of McIntosh et al. [9], the various treatments are provided by different therapists to avoid contamination.

Another noteworthy aspect of the study is the choice of treatment-as-usual as a control condition. This was done for several reasons. Studies that imposed a wait-list control condition in AN patients reported drop-out rates of up to 70% [26]. In another trial, a control arm that used dietary advice had a 73% overall treatment failure rate [25]. Due to the ethical problem of denying active treatment to AN patients, and considering the fact that the health care system in Germany enables every AN patient to get both in- and outpatient psychotherapy, it appeared to be advisable to choose TAU as a control condition. With this choice, the study faces the problem that therapeutic interventions in the TAU-condition could be quite heterogeneous. In order to give consideration to this heterogeneity, the treatment dosage and approaches used in the TAU-condition is documented in detail.

Specific challenges encountered by this outpatient study were the definition of the outline and the boundaries of treatment. In Germany, as in most health care systems, patients with manifest AN are regularly offered inpatient treatments – amongst others – to cope with potential medical complications. In the ANTOP study, we specified that a BMI over 15 was necessary for participation; also, that either a drop of BMI below 14 or the occurrence of medical complications would allow inpatient treatment in regard to crisis intervention for up to four weeks as part of the study protocol. This specification is the conclusion of the results of previous studies that demonstrated the necessity of developing treatment protocols that include dealing with medical difficulties without dropping patients from the protocol to which they were assigned [27].

The principal investigators and leaders of the participating centres of this particular study are, clinically speaking, very experienced in the treatment of patients with AN. We were therefore fully aware that in conducting this study we would have to face numerous difficulties such as cancellation of some therapy sessions, patient-initiated changes of settings, drop-out rates, and medical complications. We were also aware that many patients would not be cured after the 10 months of outpatient treatments. Nevertheless, this is the first AN study where numerous methodological disadvantages were excluded from the outset. As a result, it will improve the evidence-based knowledge about outpatient treatment efficacy in patients with AN.

## Competing interests

The authors declare that they have no competing interests.

## Authors' contributions

SZ is the principal investigator of the study, WH is the co-investigator. BW is the leading biostatistician. SZ, WH, and BW conceived of the study. They made substantial contributions to concept development, design, manuals and methodology. HCF and GG participated in the coordination of the study and the development of the manuals, and drafted sections of the manuscript. KEG, MT, and HS also participated in the coordination of the study and the development of the manuals. MdZ is the network coordinator and participated in the design of the study and the development of the manuals. CSB and HS made substantial contributions to preparation and methodology of the study protocol. All authors read and approved the final manuscript.
